# The Sexual Instinct; Its Use and Dangers as Affecting Heredity and Morals

**Published:** 1899-04

**Authors:** 


THE SEXUAL INSTINCT; Its Use and Dangers as Affecting Heredity and Morals. By JAMES FOSTER SCOTT, B. A. (Yale University), M. D., C. M. (Edinburgh University); Late Obstetrician to Columbia Hospital for Women and Lying-in Asylum, Washington, D. C., etc. Octavo, pp. 436. New York: E. B. Treat & Co. 1899. Price, $2.00.

The plain talk contained in this book needs no defense, the times with the present tendency to loose morals fully justify the deep searching which the author has made with his free lancet.

The design of the work is to furnish the nonprofessional man with a sufficiently thorough knowledge of matters pertaining to the sexual sphere—knowledge which he cannot afford to be without. To the professional man the work offers features not to be despised or turned aside, but which in his capacity as family adviser will be of inestimable value to him.

In fairness to the author it must be stated that his knowledge of these subjects has been acquired through legitimate channels. Upon his very entrance into university life his attention was first directed to the subject by an address from the late President Porter of Yale University; then came the experience as a medical student at Edinburgh, Vienna and London; then a residence of two and a half years in a hospital devoted exclusively to obstetrics and the diseases of women, followed by several years more of hospital and private practice.

Thus as the author states he has learned to appreciate and respect the rôle of woman in nature, and to abhor the ignorance which will permit men to throw aside the elements of their manhood—veracity, cleanliness, health and fitness for ancestorship.

The author divides the work into thirteen chapters, among the most important being Chapter I., introductory; the sexual instinct and the importance of a just appreciation of its influence. Chapter II., the physiology of sexual life. Chapter III., a proper calculation of the consequences of impurity from the personal standpoint. Chapter IV., woman, and the unmanliness of degrading her. Chapter V., some of the influences which incite to sexual immorality. Chapter VI., prostitution and the influences that lead a woman into such a life. Chapter VII., the regulation of prostitution.

The remaining chapters deal with the venereal diseases as affecting particularly the female sex and show the dangers of the different ailments produced not only upon those directly affected, but also upon the coming generations.

In make-up the book is similar to others of the well known firm of E. B. Treat & Co., which is sufficient guarantee of its excellence.

